# Poster Session I - Poster of Distinction I - A156 RECURRENCE RATES FOLLOWING ENDOSCOPIC MUCOSAL RESECTION (EMR) VERSUS ENDOSCOPIC SUBMUCOSAL DISSECTION (ESD) FOR COLORECTAL POLYPS: A SYSTEMATIC REVIEW AND META-ANALYSIS OF RANDOMIZED CONTROLLED TRIALS

**DOI:** 10.1093/jcag/gwaf042.156

**Published:** 2026-02-13

**Authors:** H Li, T Nishimura, K Khalaf, M A Bucheeri, Y Yuan, N Calo, G May, J Mosko, C W Teshima

**Affiliations:** Medicine, Div of Gastroenterology, St Michael’s Hospital, Toronto, ON, Canada; Medicine, Div of Gastroenterology, St Michael’s Hospital, Toronto, ON, Canada; Medicine, Div of Gastroenterology, St Michael’s Hospital, Toronto, ON, Canada; Medicine, Div of Gastroenterology, St Michael’s Hospital, Toronto, ON, Canada; McMaster University, Hamilton, ON, Canada; Medicine, Div of Gastroenterology, St Michael’s Hospital, Toronto, ON, Canada; Medicine, Div of Gastroenterology, St Michael’s Hospital, Toronto, ON, Canada; Medicine, Div of Gastroenterology, St Michael’s Hospital, Toronto, ON, Canada; Medicine, Div of Gastroenterology, St Michael’s Hospital, Toronto, ON, Canada

## Abstract

**Background:**

Colorectal cancer is among the most common cancers worldwide, and the majority of these malignancies originate from adenomatous polyps. EMR and ESD are widely used for resecting large, laterally spreading colorectal polyps. While ESD offers higher en bloc resection rates, its comparative efficacy and safety relative to EMR remain uncertain, particularly in Western practice.

**Aims:**

This study aimed to evaluate the recurrence and adverse event rates of EMR versus ESD in adult patients with colorectal polyps ≥20 mm.

**Methods:**

We conducted a systematic review and meta-analysis of RCTs comparing EMR and ESD for non-pedunculated colorectal polyps ≥20 mm. MEDLINE, Embase and Cochrane CENTRAL were searched from inception to April 2025. Eligible studies were required to report at least one relevant clinical outcome. The primary outcome was recurrence rate; secondary outcomes included total adverse events, bleeding, and perforation. Certainty of evidence was assessed using the GRADE framework.

**Results:**

In this meta-analysis of three randomized controlled trials including 484 patients, ESD was associated with significantly lower recurrence rates compared to EMR (OR 3.24, 95% CI: 1.58–6.64), with high certainty of evidence. There were no significant differences between EMR and ESD in total adverse events (OR 1.14, 95% CI:0.78–1.66), bleeding (OR 0.97, 95% CI: 0.53–1.76), or perforation rates (OR 1.01, 95% CI: 0.14–7.22), though certainty for these outcomes was moderate due to heterogeneity and imprecision.

**Conclusions:**

ESD is associated with significantly lower recurrence rates compared to EMR in the treatment of large colorectal polyps. These findings support the use of ESD when recurrence risk is a primary concern, while EMR remains a feasible alternative with comparable safety.

**Funding Agencies:**

None

